# A Protein Complex Containing the Conserved Swi2/Snf2-Related ATPase Swr1p Deposits Histone Variant H2A.Z into Euchromatin

**DOI:** 10.1371/journal.pbio.0020131

**Published:** 2004-03-23

**Authors:** Michael. S Kobor, Shivkumar Venkatasubrahmanyam, Marc D Meneghini, Jennifer W Gin, Jennifer L Jennings, Andrew J Link, Hiten D Madhani, Jasper Rine

**Affiliations:** **1**Department of Molecular and Cell Biology, University of CaliforniaBerkeley, CaliforniaUnited States of America; **2**Department of Biochemistry and Biophysics, University of CaliforniaSan Francisco, CaliforniaUnited States of America; **3**Department of Microbiology and Immunology, Vanderbilt University School of MedicineNashville, TennesseeUnited States of America

## Abstract

The conserved histone variant H2A.Z functions in euchromatin to antagonize the spread of heterochromatin. The mechanism by which histone H2A is replaced by H2A.Z in the nucleosome is unknown. We identified a complex containing 13 different polypeptides associated with a soluble pool of H2A.Z in Saccharomyces cerevisiae. This complex was designated SWR1-Com in reference to the Swr1p subunit, a Swi2/Snf2-paralog. Swr1p and six other subunits were found only in SWR1-Com, whereas six other subunits were also found in the NuA4 histone acetyltransferase and/or the Ino80 chromatin remodeling complex. H2A.Z and *SWR1* were essential for viability of cells lacking the *EAF1* component of NuA4, pointing to a close functional connection between these two complexes. Strikingly, chromatin immunoprecipitation analysis of cells lacking Swr1p, the presumed ATPase of the complex, revealed a profound defect in the deposition of H2A.Z at euchromatic regions that flank the silent mating type cassette *HMR* and at 12 other chromosomal sites tested. Consistent with a specialized role for Swr1p in H2A.Z deposition, the majority of the genome-wide transcriptional defects seen in *swr1*Δ cells were also found in *htz1*Δ cells. These studies revealed a novel role for a member of the ATP-dependent chromatin remodeling enzyme family in determining the region-specific histone subunit composition of chromatin in vivo and controlling the epigenetic state of chromatin. Metazoan orthologs of Swr1p (*Drosophila* Domino; human SRCAP and p400) may have analogous functions.

## Abstract 2

The histone variant H2A.Z localizes specifically to euchromatin and prevents spreading of silent chromatin into adjacent euchromatin. This study shows that the chromatin-remodeling complex SWR1-Com helps deposit H2A.Z onto euchromatin in yeast

## Introduction

Histones are the major constituent of chromatin and exert a profound influence on most if not all aspects of chromosome behavior. The functional state of chromatin is regulated, in part, by histone modifying enzymes and ATP-dependent chromatin remodeling enzymes. Members of the latter enzyme class alter the structure of nucleosomes or slide them along DNA in vitro (reviewed in Becker and Horz 2002; Peterson 2002). These enzymes have a catalytic DNA-dependent ATPase subunit, which is similar in sequence to those of the DEAD/DEAH-box class of RNA-dependent ATPases. The prototype for this family is the Saccharomyces cerevisiae Swi2/Snf2 protein, originally identified for its role in promoting transcription.

In addition to histone modification and nucleosome remodeling/sliding, there is a third form of chromatin regulation that involves the replacement of canonical histones with histone variants. For example, replacement of histone H3 by a conserved H3 variant (called Cse4p in *S. cerevisiae,* Cid in *Drosophila,* or CENP-A in humans) is essential for the assembly of the kinetochore (reviewed in Smith 2002). The other histone variant that is conserved between yeast and humans is H2A.Z, which replaces H2A in about one in ten nucleosomes. By convention, the gene encoding H2A.Z in *Saccharomyces* is referred to as *HTZ1* and mutant forms of the gene are referred to as *htz1.* We have shown previously that an important function of H2A.Z in S. cerevisiae is to prevent the spreading of silent chromatin, also termed heterochromatin, into adjacent euchromatic regions (Meneghini et al. 2003). Silencing in S. cerevisiae occurs at the *HMR* and *HML* silent mating type cassettes, near telomeres, and in the rDNA (reviewed in Rusche et al. 2003). All three types of silencing require the NAD-dependent histone deacetylase Sir2p. Telomeric and *HM* silencing also require the histone H3/H4 tail binding proteins, Sir3p and Sir4p. In yeast cells lacking H2A.Z, the Sir complex spreads beyond its normal boundaries at *HMR* and into neighboring euchromatin, resulting in the repression of gene expression (Meneghini et al. 2003). This repression is reversed by a deletion of *SIR2* or a deletion of the nucleation sites for silencing at *HMR*. Likewise, the silencing of genes near telomeres in *htz1*Δ cells is reversed by a deletion of *SIR2*. In yeast, H2A.Z itself is enriched in the euchromatic region flanking *HMR* and is depleted in silent regions. Genetic analysis indicates that H2A.Z acts independently of a characterized chromatin boundary element that occurs on the right border of the *HMR* silent cassette. Thus, H2A.Z is a euchromatin-specific factor that antagonizes the spread of silencing through a mechanism that is independent of at least one characterized boundary element (Meneghini et al. 2003). However, the creation of a boundary for the spread of silenced chromatin likely involves additional protein factors, such as the double bromodomain protein, Bdf1p, whose function is similar to that of H2A.Z and which binds preferably to acetylated histones that are found in euchromatin outside of silenced regions (Ladurner et al. 2003; Matangkasombut and Buratowski 2003).

Despite its critical role in preventing the spread of heterochromatin, the mechanism by which H2A.Z is deposited in euchromatin is unknown. The canonical histones can be deposited by both replication-coupled and replication-independent deposition mechanisms (reviewed in Haushalter and Kadonaga 2003). In human cells, the replication-coupled deposition pathway is essential for progression through S-phase and for cell viability (Hoek and Stillman 2003; Ye et al. 2003). In contrast, in budding yeast, no single deposition pathway is essential for viability (Kaufman et al. 1998; Formosa et al. 2002). For example, the histone H3/H4 chaperones CAF-I and Asf1p function synergistically during replication-coupled histone deposition in vitro and cooperate to form heterochromatin in vivo. However, neither CAF-I nor Asf1p is essential for cell viability in *S. cerevisiae,* and mutants lacking both proteins are also viable (Tyler et al. 1999). Nap1p, a yeast homolog of a mammalian histone chaperone purified on the basis of a replication-independent assembly assay, is also dispensable for viability in S. cerevisiae (Ishimi and Kikuchi 1991; Kellogg and Murray 1995). Thus, other mechanisms must operate to deposit chromatin in living cells. One candidate is the *Drosophila* factor ACF and the orthologous human complex RSF, which each contain a ISWI-type Swi2/Snf2 ATPase subunit (reviewed in Haushalter and Kadonaga 2003). These factors promote the ATP-dependent assembly of ordered nucleosome arrays in vitro, but their precise in vivo roles have not been firmly established.

Even less is known about the mechanisms of deposition of variant histones. Understanding the mechanism by which euchromatin that contains H2A.Z is formed requires the identification of the machinery that catalyzes its deposition. The results of this study identify a multisubunit protein complex, SWR1-Complex (SWR1-Com), which contains a Swi2/Snf2 paralog and is required for H2A.Z deposition and function in S. cerevisiae. We link this complex structurally and genetically to the NuA4 histone acetyltransferase (HAT) and the Ino80-C chromatin remodeling complex.

## Results

### A Protein Complex (SWR1-Com) Associated with the Histone Variant H2A.Z

**Figure 1:**
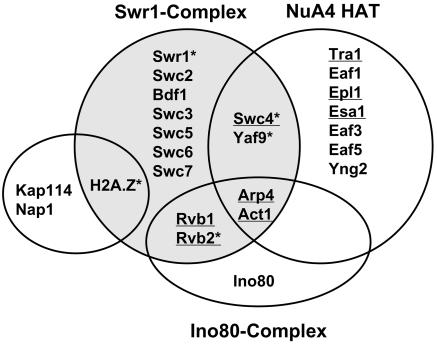
Subunit Architecture of SWR1-Com and Overlap with NuA4 and Ino80-C Complexes Venn diagram showing proposed subunit compositions of the SWR1, NuA4, Ino80p-C, and Nap1p/Kap114p complexes. Assignments were based on the data shown in Table 1 and Figure 2 and Figure 3. Proteins used in TAP purifications are indicated by “*” and proteins encoded by essential genes are underlined.

H2A.Z is important for specifying euchromatic regions in the genome of S. cerevisiae (Meneghini et al. 2003). To determine which other proteins contribute to directing H2A.Z to its chromosomal locations, we purified proteins associated with a soluble pool of H2A.Z from whole cell extracts of a yeast strain harboring an allele of *HTZ1* that encodes a carboxyl-terminal fusion to the tandem affinity purification (TAP) tag (see Materials and Methods) (Rigaut et al. 1999). These initial purifications were performed under low salt conditions and with limited wash steps to maximize protein complex recovery, with more stringent conditions used subsequently to distinguish strong from weak and potentially artifactual interactions (see below). The protein compositions of the samples were determined using Direct Analysis of Large Protein Complexes methodology, which consists of tryptic digestion of the mixture, multidimensional microcapillary chromatography, tandem mass spectrometry, and genome-assisted analysis of the acquired spectral data (Link et al. 1999; Sanders et al. 2002). A protein was judged to be associated with H2A.Z if the number of corresponding peptides in the H2A.Z-TAP purified material was higher than in the material purified from strains lacking a tagged H2A.Z protein and if the protein passed additional criteria described below. Proteins established to nonspecifically copurify with TAP-tagged proteins were excluded from the analysis (Shevchenko et al. 2002). Using these criteria and additional purifications (described below), we identified 15 proteins associated with H2A.Z (Table 1), of which 13 form a complex designated SWR1-Com (Figure 1 and see below). The largest subunit corresponded to Swr1p (Swi2/Snf2-related), an uncharacterized member of the Swi2/Snf2 family of ATP-dependent chromatin remodeling enzymes (Pollard and Peterson 1998).

**Table 1:**
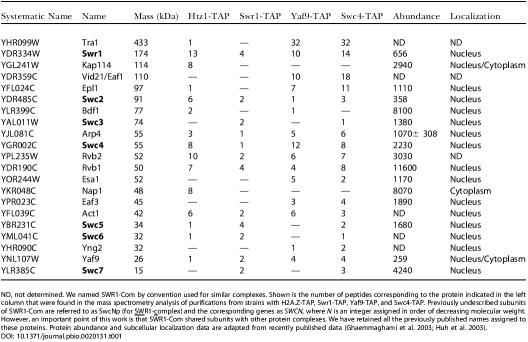
Peptides in TAP Purifications

ND, not determined. We named SWR1-Com by convention used for similar complexes. Shown is the number of peptides corresponding to the protein indicated in the left column that were found in the mass spectrometry analysis of purifications from strains with H2A.Z-TAP, Swr1-TAP, Yaf9-TAP, and Swc4-TAP. Previously undescribed subunits of SWR1-Com are referred to as SwcNp (for SWR1-complex) and the corresponding genes as *SWCN,* where *N* is an integer assigned in order of decreasing molecular weight. However, an important point of this work is that SWR1-Com shared subunits with other protein complexes. We have retained all the previously published names assigned to these proteins. Protein abundance and subcellular localization data are adapted from recently published data (Ghaemmaghami et al. 2003; Huh et al. 2003)

### SWR1-Com Shared Subunits with the Essential HAT NuA4 and the Ino80-C Chromatin Remodeling Complex

Four SWR1-Com subunits that are also found in the Ino80-C chromatin remodeling complex are Rvb1p, Rvb2p, Arp4, and actin (Shen et al. 2000). Similarly, Yaf9p, as shown below and by others (Le Masson et al. 2003), as well as Arp4p and actin, are also components of the NuA4 HAT (Galarneau et al. 2000). To determine whether the proteins that associated with H2A.Z formed one discrete complex, multiple complexes, or were copurifying contaminants, three of these proteins were themselves tagged with the TAP domain. Complexes from the soluble fraction of whole cell extracts were purified in conditions similar to those used for the H2A.Z-TAP purification, and the composition of the purified material was evaluated by the same procedure used for the H2A.Z-TAP (summarized in Table 1 and Figure 1). With the exception of the histone chaperone Nap1p and the import protein Kap114p, the proteins that copurified with TAP-tagged Swr1p were similar to the set found with H2A.Z, except that two additional proteins, designated here as Swc3p and Swc7p, were identified. Similarly, purifications from strains with TAP-tagged Swc4p and Yaf9p yielded nearly all the proteins associated with Swr1p and H2A.Z and lacked Nap1p and Kap114p. Like the Swr1-TAP material, the Swc4-TAP-associated material contained Swc3p and Swc7p, supporting the assignment of these two proteins to SWR1-Com. TAP-tagged Swc4p and Yaf9p also associated with most of the subunits of the NuA4 complex (including Tra1p, Epl1p, Eaf3p, Yng2p, and the catalytic subunit Esa1p). These data suggested that SWR1-Com and NuA4 shared the Yaf9p, Swc4p, Arp4, and actin subunits.

**Figure 2:**
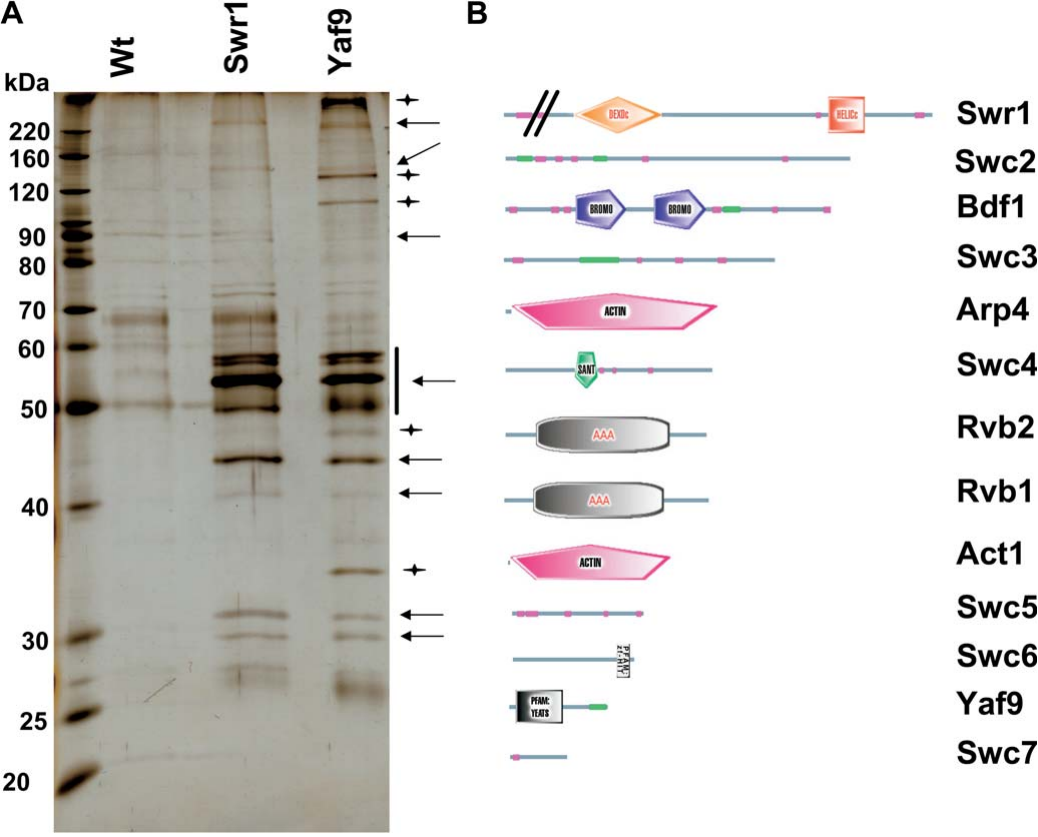
SWR1-Com Shared Subunits with NuA4 and Contained Proteins with Motifs Involved in Chromatin Biology (A) Protein complex overlap. Purifications were performed under high stringency conditions (see Materials and Methods) from Swr1-TAP, Yaf9-TAP, and untagged control strains, resolved by SDS-PAGE and stained with silver. Due to the relatively low efficiency of the Swr1-TAP purification, the wt and Swr1-TAP purifications were performed from twice the amount of starting material compared to Yaf9-TAP. Not all proteins identified by mass spectrometry were clearly visible on the gel. Arrows point to proteins that were common to the Swr1-TAP and Yaf9-TAP purifications, whereas stars point to proteins that were found only in the Yaf9-TAP purifications as judged by visual inspection and comparison of protein sizes with the data deduced from mass spectrometry. The vertical bar indicates that proteins in that area of the gel could not be clearly resolved. (B) Domain structure of SWR1-Com. Shown are SMART domain representations of individual proteins assigned to the SWR1-Com taken from the SMART database (http://smart.embl-heidelberg.de/). Domain names are included, green bars indicate coil-coiled regions, and magenta bars indicate regions of low complexity. The amino-terminal part of Swr1p is not to scale.

Representative complex purifications under high stringency conditions (see Materials and Methods) from strains with either the Swr1-TAP, Yaf9-TAP, or an untagged control strain are shown in Figure 2A. Proteins that copurified with both Swr1-TAP and Yaf9-TAP represented subunits of SWR1-Com (Figure 2A, arrows), whereas proteins that only copurified with Yaf9-TAP represented specific NuA4 subunits (Figure 2A, stars). A schematic representation of the domain structures of SWR1-Com subunits is presented in Figure 2B. Several of the proteins in the complex contained motifs (SANT, Bromo, YEATS, and HIT) found in proteins associated with chromatin, suggesting that SWR1-Com acts directly on chromatin.

Since the histone chaperone Nap1p and the import factor Kap114p copurified with H2A.Z but not other members of the complex, they were likely to be part of an H2A.Z-containing protein complex distinct from SWR1-Com. Affinity purification of TAP-tagged Rvb2p, an established component of the Ino80-C chromatin remodeling complex, yielded peptides corresponding to the other known subunits of the Ino80-C complex as well as six members of SWR1-Com, three of which (Swc4p, Arp4p, and actin) are also subunits of NuA4 (Table S1). Consistent with the assignment of SWR1-Com subunits, a percentage of the cellular pool of these proteins cosedimented with each other upon glycerol gradient centrifugations of whole cell extracts (Figure S1).

**Figure 3:**
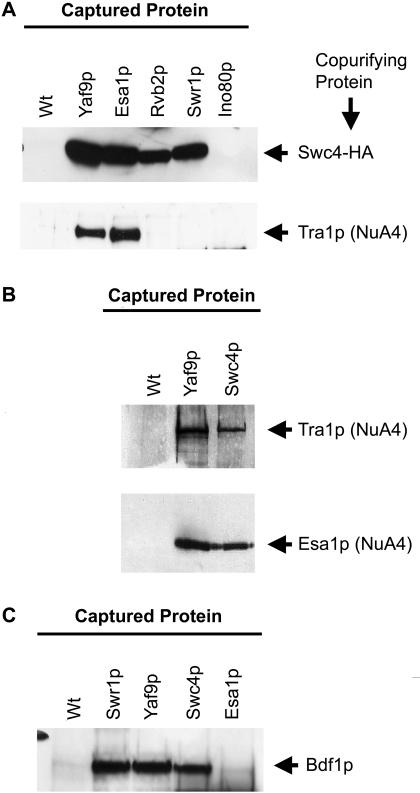
Swc4p and Bdf1p Were Components of SWR1-Com This figure shows immunoblots of analytical-scale TAP purifications. The captured TAP-tagged protein is indicated above the gels, and the protein that was tested for association is indicated at the right side. (A) Association of Swc4p and Tra1p. Swc4-HA was present in purifications from Yaf9-TAP, Esa1-TAP, Rvb2-TAP, and Swr1-TAP but not Ino80-TAP. NuA4 was only present in the Yaf9-TAP and Esa1-TAP material. (B) Reciprocal confirmation of Swc4p being part of NuA4. Swc4-TAP and Yaf9-TAP purified material contained NuA4 components Esa1p and Tra1p. (C) Association of Bdf1p. Bdf1p was present in purifications from Swr1-TAP, Yaf9-TAP, and Swc4-TAP but not Esa1-TAP.

The initial purifications suggested that Swc4p and Bdf1p, both of which have domains that are involved in recognition of histone tails, copurified with H2A.Z-TAP and might be part of SWR1-Com. Independent assessment of the composition of the complexes deduced by mass spectrometry was obtained by analytical-scale affinity purifications of Yaf9-TAP, Esa1-TAP, Rvb2-TAP, Swr1-TAP, and Ino80-TAP from cells containing a version of Swc4p that was fused at its carboxyl-terminus to a triple hemagglutinin (HA) tag. These analytical-scale affinity purifications were more stringent than the initial TAP purifications and therefore served to eliminate false-positive results and to provide independent tests of interactions. Anti-HA epitope antibodies and antibodies against Tra1p, the largest subunit of NuA4, were used to analyze the copurified material. Both Yaf9-TAP and Esa1-TAP associated with comparable amounts of Tra1p and Swc4p, supporting the assignment of Yaf9p and Swc4p as new subunits of NuA4. Likewise, Rvb2-TAP and Swr1-TAP copurified with a substantial amount of Swc4-HA, but Ino80-TAP did not. Rvb2-TAP, Swr1-TAP, and Ino80-TAP were not associated with Tra1p (Figure 3A). These data were consistent with Swr1p and Rvb2p being components of SWR1-Com and not of NuA4. Further supporting the assignment of Swc4p as a subunit of NuA4, significant amounts of the NuA4 subunits Tra1p and Esa1p were present in material from Swc4-TAP analytical-scale purifications (Figure 3B).

The number of peptides corresponding to Bdf1p in the TAP purifications was low, and Bdf1p was found only in the H2A.Z-TAP and the Yaf9-TAP (Table 1). Bdf1p's potential presence was tested further by additional analytical-scale affinity purifications from strains carrying Yaf9-TAP, Swr1-TAP, Swc4-TAP, and Esa1-TAP and immunoblotting with an antibody against Bdf1p. Bdf1p associated with Swr1-TAP, Yaf9-TAP, and Swc4-TAP but not with Esa1-TAP or untagged control material, supporting the assignment of Bdf1p as a subunit of SWR1-Com (Figure 3C).

### SWR1-Com Selectively Associated with Histone H2A.Z Versus H2A

To determine whether subunits of SWR1-com associated specifically with H2A.Z or both H2A.Z and H2A, TAP-tagged versions of H2A.Z and H2A were purified from cells containing HA-tagged versions of the five different SWR1-Com components Swr1p, Swc2p, Swc3p, Swc4p, and Swc7p, and the nuclear import factor Kap114p. The composition of the copurifying material was then evaluated by immunoblotting with antibodies against the HA tag, Bdf1p, and Tra1p. Yaf9-TAP served as a positive control for recovery of SWR1-Com and NuA4.

**Figure 4:**
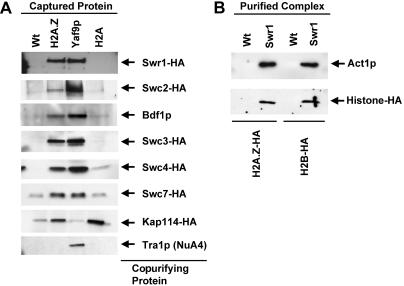
SWR1-Com Associated Selectively with H2A.Z and Contained H2B (A) Analytical-scale TAP purifications from H2A.Z-TAP, Yaf9-TAP, and H2A-TAP were analyzed by immunoblotting for the components indicated on the right. SWR1-Com preferentially associated with H2A.Z-TAP, whereas Kap114-HA associated equally with H2A.Z-TAP and H2A-TAP but not Yaf9-TAP. (B) SWR1-Com was purified from strains with HA-tagged versions of either H2A.Z or H2B and analyzed by immunoblotting for the presence of these histones as well as the SWR1-Com subunit Act1p.

H2A.Z associated with a substantial fraction of the SWR1-Com as judged by the comparable intensity of the signal for SWR1-Com subunits in the material copurified with H2A.Z-TAP and Yaf9-TAP, whereas no NuA4 copurified with H2A.Z-TAP based upon the absence of Tra1p in the H2A.Z-TAP sample (Figure 4A). In contrast, histone H2A copurified with only trace amounts of Swc2-HA, Swc3-HA, Swc4-HA, Swc7-HA, and Bdf1p and with virtually no Swr1-HA (see Figure 3). Kap114-HA associated with both H2A.Z-TAP and H2A-TAP but not Yaf9-TAP, suggesting that it did not discriminate canonical and variant H2A. Hence, based on the apparent relative strength of the interactions, SWR1-Com (in contrast to Kap114-HA) associated primarily with H2A.Z, although weak affinity of SWR1-Com to H2A was possible. Furthermore, these experiments also supported the assignment of Swc3p and Swc7p to SWR1-Com despite peptides for these two proteins being present only in the initial Swr1-TAP and Swc4-TAP purifications.

Canonical H2A that is not bound to chromatin is usually found in a H2A/H2B dimer (Jackson 1987). The presence of H2B in the SWR1-Com was investigated by purifying SWR1-Com from strains containing H2A.Z-HA and H2B-HA. SWR1-Com contained H2A.Z-HA and also H2B-HA (Figure 4B). These data raised the possibility that this complex used H2A.Z/H2B dimers as a substrate.

### Similar Gene Expression Profiles of *htz1*Δ and *swr1*Δ Cells

**Figure 5:**
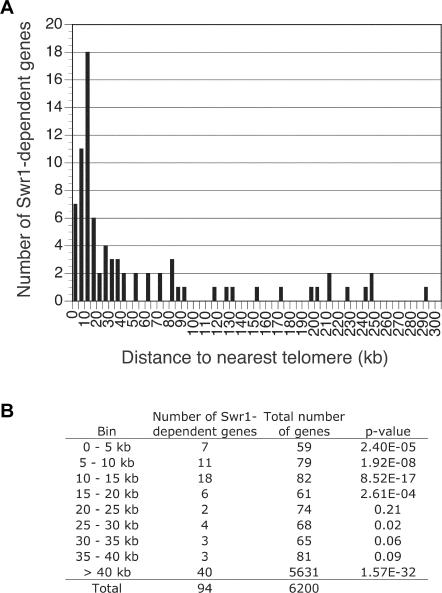
Chromosomal Distribution of Swr1p-Activated Genes (A) Histogram showing the number of Swr1p-activated genes as a function of their distance to the nearest chromosome end. (B) The statistical significance of the enrichment of Swr1p-activated genes as a function of distance to the nearest telomere, and the significance of the depletion of Swr1p-activated genes in regions greater than 40 kb from a telomere, were determined using the hypergeometric function (Tavazoie et al. 1999).

To determine the extent to which the role of H2A.Z depends upon SWR1-Com function, genome-wide transcription profiles of *swr1*Δ cells were compared to the profiles of *htz1*Δ cells (Meneghini et al. 2003). To permit an optimal comparison, experiments were performed under the conditions used previously to analyze *htz1*Δ cells (see Materials and Methods). Due to the role of H2A.Z in anti-silencing, H2A.Z-dependent genes tend to be located near silenced domains such as telomeres. This theme was echoed in the results from the *swr1*Δ mutant. Specifically, 42 of the 94 (45%) Swr1p-dependent genes were within 20 kb of a chromosome end, which is less than 3% of the genome (Figure 5A). This enrichment is highly significant, as judged by *p*-values estimated from the hypergeometric distribution (Figure 5B). Swr1p-dependent genes were underrepresented from regions more than 40 kb from a telomere, suggesting that, as seen earlier for H2A.Z, the telomere-proximal genes were most sensitive to loss of Swr1p function.

**Figure 6:**
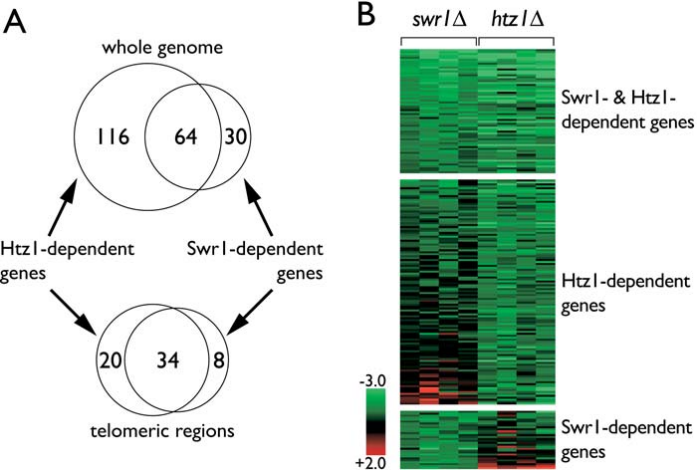
Relationship of Genes Activated by Swr1p or H2A.Z (A) The Venn diagram of number of genes that exhibited a significant decrease in expression in *swr1*Δ cells (this work) or *htz1*Δ cells (Meneghini et al. 2003), revealing a large overlap. Shown on the top is the relationship for the genome overall and on the bottom for genes within 20 kB of a telomere. H2A.Z-dependent genes whose expression could not be determined in *swr1*Δ cells were omitted. (B) A color representation of all genes that were significantly reduced in expression in *swr1*Δ cells only, *htz1*Δ cells only, or both, grouped according to (A). Each column represents data from an independent microarray experiment that compared genome-wide expression in mutant cells of the indicated genotype to wt cells. Each row represents the changes in expression of a single gene across the eight experiments. Change in expression measured as the log_2_ of the mutant/wt expression ratio is indicated according to the color scale shown. Red cells refer to genes found to have increased expression in either *swr1*Δ cells or *htz1*Δ cells that decreased in expression in the other mutant. Excluded from representation are genes that increased expression in both mutants.

Comparison of the transcript profile across the genome of *swr1Δ* cells to that of *htz1*Δ cells also revealed a marked overlap (Figure 6A). Ninety-four genes displayed reduced expression in the *swr1*Δ mutant compared to wild type. Of these 94 Swr1p-dependent genes, 64 were also reduced in expression in *htz1*Δ (Figure 6A). This remarkably large overlap is highly significant (*p* = 2.9 × 10^−80^, calculated using the hypergeometric distribution) and even more impressive for telomere-proximal genes. These data suggested that Swr1p and H2A.Z shared a common function in regulating gene expression.

A substantial number of H2A.Z-dependent genes (116) did not appear to require Swr1p for expression. A color representation of the *swr1*Δ and *htz1*Δ datasets grouping the genes described in Figure 6A (Figure 6B) revealed that a subset of these appeared to have mildly reduced expression levels in *swr1*Δ cells but were not reduced enough to meet the stringent significance cutoff. However, there also were clear examples of genes that required H2A.Z for expression but not Swr1p. Likewise, there were clear examples among the 94 genes that required Swr1p for expression but did not require H2A.Z (Figure 6).

### Swr1p Was Required for H2A.Z Deposition In Vivo

The evidence linking H2A.Z and Swr1p function and the association of both H2A.Z and H2B with SWR1-Com suggested that SWR1-Com was responsible for depositing H2A.Z onto chromatin in vivo, perhaps in the form of an H2A.Z/H2B dimer. (The Swr1p relatives in the ACF and RSF complexes perform related roles in assembling chromatin in vitro (reviewed in Haushalter and Kadonaga 2003). If so, then cells lacking Swr1p should display reduced levels of H2A.Z in chromatin.

**Figure 7:**
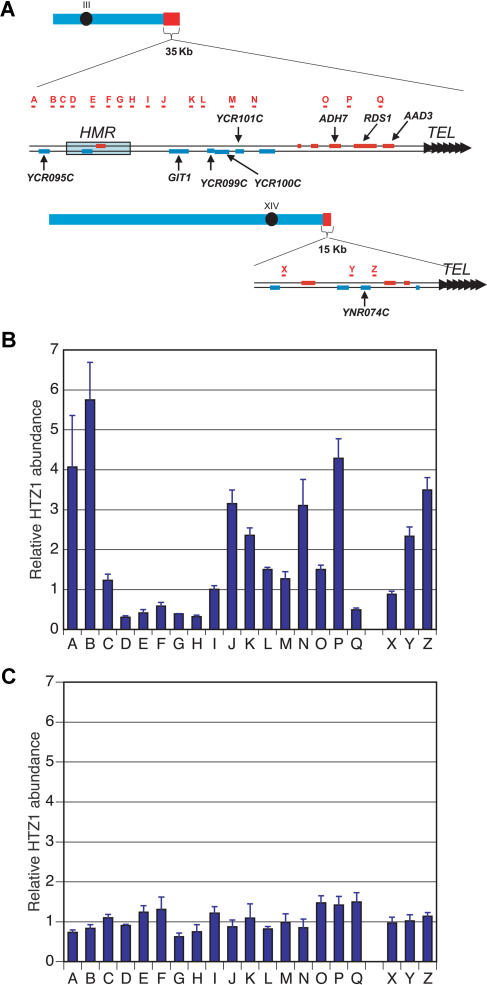
ChIP Analysis of HA3-H2A.Z Deposition in the *HMR* Region and Near the Right Telomere of Chromosome XIV (A) Location of PCR primers. (B) ChIP results in wild type (bars indicate relative enrichment versus a probe in the *PRP8* ORF; standard errors are shown). The ChIP enrichment signal at *HMR* relative to *PRP8* being less than 1.0 indicated some H2A.Z deposition occurred at the *PRP8* control region. (C) ChIP results in *swr1*Δ cells.

To test this prediction, we performed chromatin immunoprecipitation (ChIP) experiments comparing wild type to *swr1*Δ strains expressing a functional amino-terminal triple-HA-tagged version of H2A.Z expressed from the *HTZ1* promoter at the normal chromosomal locus (HA3-H2A.Z). Consistent with H2A.Z being in a stable complex with Swr1p that protected it from protein degradation, the level of HA3-H2A.Z in *swr1*Δ strains was reduced approximately 2- to 3-fold (Figure S2). To normalize the signals from each experimental locus assayed, we measured the levels of DNA derived from a control locus whose expression is H2A.Z independent (the middle of the *PRP8* open reading frame [ORF]) in samples derived from each whole cell extract and precipitate (see Materials and Methods). We first examined HA3-H2A.Z levels at two chromosomal regions where H2A.Z prevents the spread of Sir-dependent silencing: one flanking the silent mating locus *HMR* and another near the telomere on the right arm of chromosome XIV (Figure 7A). In wild type, H2A.Z was present at levels similar to those described previously (Figure 7B); HA3-H2A.Z was depleted from the silenced *HMR* locus and enriched in the flanking euchromatic regions (Figure 7B). In addition, HA3-H2A.Z was depleted from the most telomere-proximal locus tested, *AAD3,* presumably because of telomeric silencing of this gene. Likewise, HA3-H2A.Z was highly enriched at the *YNR074C* gene, a telomere-proximal gene on chromosome XIV strongly protected from silencing by H2A.Z.

In striking contrast, in *swr1*Δ cells, the enrichment (relative to the *PRP8* locus) of HA3-H2A.Z at every locus tested approached a ratio of one (Figure 7C). These data were consistent with Swr1p being essential for the deposition of H2A.Z in the *HMR* region and near the right telomere of chromosome XIV. However, because the ChIP measurements were normalized to the *PRP8* locus, we considered the possibility that a uniform amount of HA3-H2A.Z remained at all chromosomal loci examined in the mutant, for instance if there was a specific increase in the association of HA3-H2A.Z with the *PRP8* locus rather than a decrease at all other loci. This possibility was discounted by the approximately 13-fold mean decrease in the ratio of DNA obtained from the pellet versus whole cell fractions in *swr1*Δ cells relative to wild type, indicating a substantial defect in the absolute chromatin association of HA3-H2A.Z in cells lacking Swr1p.

**Figure 8:**
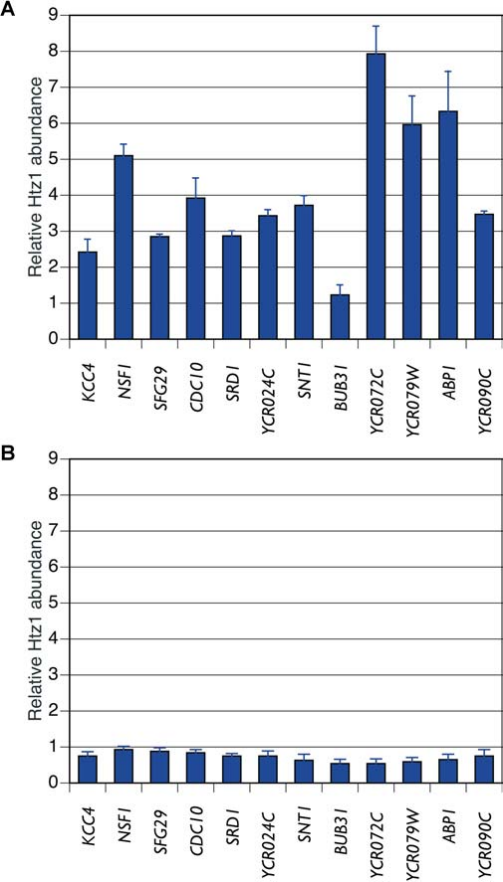
ChIP Analysis of H2A.Z Deposition at Nontelomeric Euchromatic Sites (A) ChIP results in wild type. (B) ChIP results in *swr1*Δ cells. We detected a reduced enrichment of H2A.Z at all these loci when we estimated the absolute H2A.Z abundance by dividing the amount of immunoprecipitated DNA by the amount of total input DNA for each locus.

H2A.Z is also deposited at several loci that are not near silenced regions (Meneghini et al. 2003). The function of H2A.Z at these regions is unknown. To determine if Swr1p was also required for H2A.Z deposition at such loci, we examined H2A.Z levels at 12 euchromatic regions on chromosome III that each displayed some level of deposition of HA3-H2A.Z (Figure 8A). These loci were identified in a comprehensive study of H2A.Z deposition on chromosome III (M.D.M., M. Bao, H.D.M., unpublished data). As with the regions examined above, the relative ChIP enrichment of HA3-H2A.Z approached one at each of these loci in the absence of Swr1p (Figure 8B), and the absolute amount of DNA precipitated from these loci showed a large decrease in the *swr1*Δ mutant (data not shown). Thus, Swr1p was broadly required for the deposition of HA3-H2A.Z, even in regions distant from silenced domains. It is worth noting that for several of the loci examined in the *swr1*Δ mutant, the ChIP enrichment was significantly less than one, suggesting that there may exist some residual deposition of HA3-H2A.Z at the *PRP8* locus in these cells.

### NuA4 Function Was Required for SWR1-Com to Support Cell Growth

The sharing of four proteins between the SWR1-Com and NuA4 (see Figure 1) raised the possibility that SWR1-Com was functionally linked to NuA4, which is the major HAT for histones H4 and H2A. Initial purifications from Yaf9-TAP and Swc4-TAP strains suggested that the protein encoded by the nonessential gene *YDR359C* was a subunit of NuA4, consistent with some earlier results from high-throughput studies (Gavin et al. 2002). Initial efforts to fuse a triple HA-tag to the carboxy-terminus of *YDR359C* were unsuccessful, but we noticed that all proteins encoded by *YDR359C* orthologs from the *Saccharomyces* sensu strictu strains had a carboxy-terminal extension of approximately 22 amino acids, suggesting a possible error in the S. cerevisiae sequence. Therefore, we chose to integrate a triple HA-tag at the chromosomal location that corresponded to the second-to-last codon of the sensu strictu strains and found that this version was now successfully tagged. Recently, a revised copy of *YDR359C* with the stop codon at the location we chose was deposited in GenBank and named *EAF1*. Therefore, we used this name here rather than a previously assigned name for the shorter version of *YDR359C.* Eaf1p contained a SANT domain as well as an HSA domain that is associated with SANT domains and found in helicases (Letunic et al. 2002).

**Figure 9:**
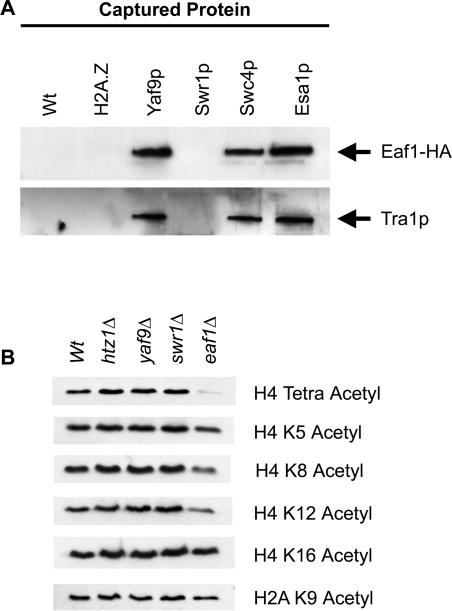
Eaf1p Was a Subunit of the NuA4 HAT (A) Eaf1-HA associated with NuA4 subunits. Immunoblots of analytical-scale TAP purifications are shown. The captured TAP-tagged protein is indicated above the gels, and the protein that was tested for association is indicated at the right. (B) Strains lacking *EAF1* have defects in histone H4 acetylation. Whole cell extracts from mutant strains indicated on the top were tested for global histone acetylation using antibodies directed against different forms of acetylated H4 and H2A as indicated on the right.

Analytical-scale affinity purifications showed that Eaf1-HA, similar to Tra1p, copurified with Yaf9-TAP, Swc4-Tap, and Esa1-TAP but not with H2A.Z-TAP and Swr1-TAP (Figure 9A). In addition, global H4 acetylation defects were evident in *eaf1*Δ but not in *htz1*Δ, *yaf9*Δ, or *swr1*Δ cells (Figure 9B). This was based on the reduced signal in immunoblot experiments obtained with an antibody directed against tetra-acetylated H4. Similar experiments with antibodies directed against individual acetylated residues revealed that the H4 acetylation defect of strains lacking *EAF1* was most profound on K8 and K12 of H4, whereas K5 and K16 of H4 were less affected (Figure 9B). Similarly, K9 of H2A did not have a strong acetylation defect in any of the mutants (Figure 9B). The physical association and the H4 acetylation defects provided independent evidence that Eaf1p was a subunit of NuA4.

**Figure 10:**
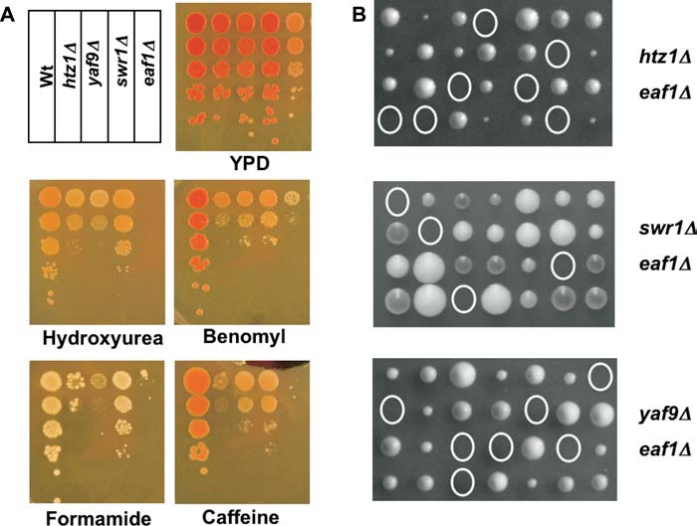
NuA4 and SWR1-Com Shared Similar Phenotypes and Interacted Genetically (A) SWR1-Com and Eaf1p were required for resistance to DNA damage and genotoxic stress. Ten-fold serial dilutions of strains from a stationary overnight culture with the indicated deletions of SWR1-Com subunits and of *EAF1* were plated and incubated at 30 °C for 2–3 d. YPD plates with the following concentrations of chemicals were used: 100 mM HU, 10 μg/ml of benomyl, 2% formamide, or 3 mM caffeine. (B) SWR1-Com and NuA4 interacted genetically. Double mutants, deduced from genetic analysis of the viable spore clones, are circled, with the two mutations of interest in each cross indicated at the side. All double mutants were inviable.

To explore genetic links between SWR1-Com and NuA4, phenotypic and double mutant analyses were performed. SWR1-Com mutants and the strain lacking *EAF1* shared sensitivities to genotoxic and stress conditions (Figure 10A). The *eaf1*Δ strains were also slow growing whereas the other strains were not. All mutant strains tested were sensitive to the DNA replication inhibitor hydroxyurea (HU) and the microtubule poison benomyl and to caffeine and formamide, reagents that elicit a number of cellular responses (Figure 10A). Strains lacking *HTZ1* and *YAF9* were comparably sensitive to HU and formamide, but *htz1*Δ strains were more sensitive to benomyl and caffeine. Strains lacking *SWR1* were less sensitive than the other strains to HU and formamide, but the sensitivity to caffeine and benomyl was comparable to that of *yaf9*Δ strains (Figure 10A). Cells lacking the NuA4 subunit Eaf1p were most sensitive to HU and caffeine, and their sensitivity to benomyl and formamide was comparable to that of *htz1*Δ mutants (Figure 10A). While the severity of the defects varied, the similar phenotypes of mutants in SWR1-Com and NuA4 suggested that the two complexes were broadly required for resistance to DNA damage and genotoxic stress.

To test whether the sensitivity to DNA damage and genotoxic stress was a shared function of SWR1-Com and NuA4, or whether these sensitivities were caused by independent functions, double mutant analysis was performed using the *EAF1* gene as an exemplary NuA4 subunit. No viable spores were obtained that had deletions of *EAF1* and *HTZ1*, *SWR1,* or *YAF9* (Figure 10B). Thus, SWR1-Com and NuA4 interacted genetically, and the two complexes shared at least one essential function.

## Discussion

Protein complexes that can substitute canonical histones with variant histones represent a fundamental mechanism for regulating the functional state of chromatin. Previous work has identified large protein complexes that assemble, remodel, and modify chromatin (reviewed in Becker and Horz 2002; Peterson 2002). In contrast, the studies described here identified a novel complex, referred to as SWR1-Com, whose putative ATPase, Swr1p, promoted the deposition of the histone H2A variant, H2A.Z, into chromatin in vivo.

### SWR1-Com, a Multisubunit Complex, Associated Specifically with H2A.Z

SWR1-Com was identified by its specific association with H2A.Z. SWR1-Com consisted of 13 subunits: six were only found in SWR1-Com, four were shared between SWR1-Com and NuA4, and four were shared between SWR1-Com and the Ino80 complex. Two subunits, Arp4 and actin, were in all three complexes (see Figure 1). Several of the subunits of SWR1-Com contained motifs highly suggestive of a role for this complex in affecting chromatin structure. Chief among these was Swr1p, a relative of the ATPase-containing subunit of the Swi2/Snf2 ATP-dependent chromatin remodeling enzyme complex (Pollard and Peterson 1998). The Swc4p subunit contained a SANT domain, suggested in other contexts to mediate association of proteins with histone tails (Boyer et al. 2002; Sterner et al. 2002). Similarly, Bdf1p contained two bromodomains that preferentially bind to acetylated tails of histones H3 and H4 (Ladurner et al. 2003; Matangkasombut and Buratowski 2003). The Swc6p subunit contained a HIT domain found in a human protein that binds to steroid receptors (Lee et al. 1995), and the Yaf9p subunit contained a YEATS domain found in several proteins involved in chromatin modification, such as the SAS-I HAT complex, and several proteins implicated in human leukemias (Xu et al. 1999; Le Masson et al. 2003). The weak interactions between SWR1-Com subunits and H2A relative to those between SWR1-Com subunits and H2A.Z suggested that the role of SWR1-Com was dedicated to those chromatin structures enriched for H2A.Z. This was further supported by the association of H2B-HA along with H2A.Z-HA with highly purified SWR1-Com, suggesting that this histone dimer was the physiological substrate for activity of SWR1-Com.

### Genome-Wide Expression Profiles and Phenotypic Analysis Identified Functional Links between H2A.Z and SWR1-Com

Similarities between the consequences of disruptions of SWR1-Com function and loss of H2A.Z protein implied that SWR1-Com was required for H2A.Z function. These similarities included the striking sensitivities of cells lacking SWR1-Com function or H2A.Z to a variety of cellular and genotoxic stresses. Comparison of the genome-wide expression profiles of *swr1*Δ and *htz1*Δ strains also revealed similar responses to loss of either function at many loci. These included silencing of genes near telomeres and the *HMR* silent mating type locus, which is antagonized by H2A.Z (Meneghini et al. 2003). In addition, there were genes distal to silenced domains that required both H2A.Z and Swr1p for their expression. Because the majority of gene expression defects seen in *swr1*Δ cells also occurred in *htz1*Δ cells, the role of Swr1p, and presumably SWR1-Com, was predominantly in promoting the function of H2A.Z.

### H2A.Z Deposition into Chromatin was Promoted by SWR1-Com

SWR1-Com promoted the deposition of H2A.Z into chromatin. At 20 sites flanking the silent *HMR* locus that were previously identified as enriched or depleted for H2A.Z, the ratio of H2A.Z at these loci relative to the *PRP8* ORF as determined by ChIP converged to unity in *swr1*Δ cells. In addition, a dramatic 13-fold decrease in the absolute enrichment of HA-H2A.Z-associated DNA was observed in *swr1*Δ cells. A similar picture emerged from the analysis of 12 sites of H2A.Z deposition across chromosome III. Therefore, Swr1p was required for enrichment of H2A.Z at a wide variety of loci, including those distal to silent regions.

Several lines of evidence suggested that Swr1p and presumably SWR1-Com play direct roles in H2A.Z deposition into chromatin. Foremost in favor of this view is the tight physical association of H2A.Z with SWR1-Com in whole cell extracts. Additionally, Swr1p, and other members of SWR1-Com, had sequence motifs found in proteins acting in chromatin and were localized in the nucleus. In particular, the Bdf1 protein via its bromodomains might be responsible for the recruitment of SWR1-Com to deposit H2A.Z to euchromatic regions, which are generally characterized by acetylation of the H4 tail. Lastly, the profound defect of *swr1*Δ cells in H2A.Z deposition, and the established actions of the Swi2/Snf2 family members directly on nucleosomes, provided further support for a direct role of Swr1p and SWR1-Com in H2A.Z deposition.

Several observations were consistent with a small amount of H2A.Z deposition in chromatin in cells lacking Swr1p function. First, some genes affected by *htz1*Δ were not affected by *swr1*Δ. Second, the enrichment of H2A.Z at some loci relative to the *PRP8* ORF was less than unity in the *swr1*Δ mutant, suggesting residual H2A.Z present at *PRP8*. Perhaps in the absence of SWR1-Com, some H2A.Z is deposited by the same mechanisms responsible for the bulk deposition of H2A. Nevertheless, the key observation was a pronounced deficiency in H2A.Z deposition in the absence of Swr1p function.

The conservation of Swr1p orthologs raises the possibility of SWR1-Com-like complexes dedicated to the deposition of variant histones in other organisms. The *Drosophila* Domino protein, human SRCAP, and human p400 are orthologs of *SWR1*, and serve as candidates for the founding members of such complexes. Mutations in *Domino* affect silencing by Polycomb proteins, although the directness of these effects is unknown (Ruhf et al. 2001). The SRCAP protein is associated with CREB-binding protein, and p400 is recruited by the Adenovirus E1A oncoprotein (Johnston et al. 1999; Fuchs et al. 2001). Although SRCAP and p400 are known primarily as transcription factors, our results suggest possible roles for these proteins in deposition of variant histones. While this work was under review, two groups independently reported on the SWR1-Com and described its role in H2A.Z deposition (Krogan et al. 2003; Mizuguchi et al. 2004). Consistent with our data, purified SWR1-Com has a Swr1p-dependent histone exchange activity (Mizuguchi et al. 2004) and hence presents a third mechanism of chromatin remodeling.

### SWR1-Com and NuA4 Function Were Linked

The SANT-domain-containing proteins Swc4p and Eaf1p were subunits of NuA4, newly described here. Both proteins associated with other NuA4 subunits, and cells lacking *EAF1* had defects in global histone H4 acetylation. Similar defects were found in a strain carrying a conditional allele of the essential *SWC4* gene (M.S.K., H.Xu, C. Boone, and J.R., unpublished data). Whereas Swc4p was shared with SWR1-Com, Eaf1p was not. However, cells lacking *EAF1* were sensitive to DNA-damaging drugs and genotoxic stress conditions, as were cells lacking subunits of SWR1-Com and H2A.Z. While NuA4's involvement in DNA damage survival was known (Bird et al. 2002; Choy and Kron 2002; Boudreault et al. 2003), the data presented here extended this view, suggesting that it might be more broadly required in the maintenance of genomic integrity in concert with SWR1-Com. Genetic interactions between *EAF1* and three SWR1-Com subunits uncover a deeper connection. Specifically, the synthetic lethality of *eafl*Δ in combination with null alleles of SWR1-Com indicated that these complexes were likely to share an essential function. That is, genes encoding subunits of SWR1-Com became essential when NuA4 activity was compromised by deletion of *EAF1,* and vice versa. While understanding the mechanisms will require further work, these data suggested important functional links between the H2A.Z deposition machinery and the NuA4 HAT.

### Why Do SWR1, NuA4, and Ino80 Complexes Share Subunits?

As discussed above, a third of the subunits of SWR1-Com are shared with the Ino80 complex, the NuA4 HAT, or both. While the sharing of subunits between different protein complexes is not unprecedented, it may reflect highly related functions, rather than vagaries of chance and circumstance in evolution. This was supported by the functional overlap and genetic interactions between SWR1-Com and NuA4. The shared subunits may act as a core scaffold, upon which the unique subunits can be assembled and exchanged during a cycle of chromatin modification. This notion finds some support in the existence of a mini-NuA4 complex, known as piccolo NuA4, which contains only some of those subunits that are unique to NuA4 (Boudreault et al. 2003). Shared subunits of SWR1-Com could coordinate the recruitment of an analogous mini-SWR1-Com to achieve histone subunit replacement, with the replacement of mini-SWR1-Com by piccolo NuA4 to achieve the acetylation of the newly reconstituted nucleosome. This model could explain why two subunits of NuA4 (Tra1 and Epl1p) were detected in the H2A.Z-associated material under low stringency conditions (see Table 1). Alternatively, the acetylation of H2A by NuA4 may facilitate its replacement by H2A.Z. Other orders of action involving the SWR1-Com, NuA4, and Ino80-C complex are also possible, such as acetylation of H2A.Z by NuA4 being a prerequisite for its exchange by SWR1-Com. Other potential roles for the sharing of subunits include targeting complexes to common locations or promoting their biogenesis or assembly. Our data may resolve an interesting paradox concerning the localization of Bdf1p on chromatin. Earlier work showed that Bdf1p is a subunit of TFIID, yet Bdf1p was found in regions where TATA box binding protein, the core subunit of TFIID, was not (Matangkasombut and Buratowski 2003). The discovery that Bdf1p is part of two distinct complexes, SWR1-Com and TFIID, explains the lack of a perfect correspondence between Bdf1p and TATA box binding protein localization.

## Materials and Methods

### 

#### Yeast techniques

Strains are listed in Table S2. Sequences encoding the TAP-tag (Rigaut et al. 1999) or a triple HA-tag (Longtine et al. 1998) were integrated in frame at the 3′ end of genes using homologous recombination and one-step gene integration of PCR-amplified modules. Similarly, complete deletion of genes was achieved by a similar strategy as described before (Longtine et al. 1998).

#### Large-scale affinity purifications

Purifications of native protein complexes were performed using extracts from strains with a segment encoding the TAP tag fused in-frame to the 3′ end of the chromosomal gene of interest (Rigaut et al. 1999). In general, purifications were performed from extracts obtained from 2 l cultures that were harvested in late logarithmic phase. Our protocol for the initial purifications presented in Table 1 was modified from published protocols in a way to maximize recovery of intact protein complexes. Briefly, cells were disrupted with a coffee grinder in the presence of dry ice pellets and resuspended in 0.8 volumes/weight of TAP-B1 (50 mM Tris-Cl [pH 7.8], 200 mM NaCl, 1.5 mM MgAc, 1 mM DTT, 10 mM NaPPi, 5 mM EGTA, 5 mM EDTA, 0.1 mM Na_3_VO_4_, 5 mM NaF, Complete Protease inhibitor cocktail [Roche, Basel, Switzerland]). Crude extracts were prepared by centrifugation in a SS34 rotor for 20 min at 14,000 rpm. These were then further clarified by ultracentrifugation (Ti70 rotor, 33,500 rpm for 60 min). NP-40 was added to a final concentration of 0.15%, and the extract was incubated with 200 μl of IgG Sepharose beads (Amersham Biosciences, Little Chalfont, United Kingdom) for 90 min at 4 °C. The beads were then washed with 800 μl of TAP-B2 (50 mM Tris-Cl [pH 7.8], 200 mM NaCl, 1.5 mM MgAc, 1 mM DTT, 0.15% NP-40). After washing, the TAP tag was cleaved by adding 10 μl of TEV protease (GIBCO, San Diego, California, United States) in 200 μl of TAP-B2 to the beads and incubating at 16 °C for 90 min. Cleaved protein complexes were eluted with an additional 200 μl of TAP-B3 (50 mM Tris-Cl [pH 7.5], 200 mM NaCl, 1.5 mM MgAc, 1 mM DTT, 4 mM CaCl_2_, 0.15% NP-40)

The material eluted by the TEV protease cleavage from the first affinity matrix was incubated with 200 μl of Calmodulin beads (Stratagene, La Jolla, California, United States) for 60 min at 4 °C. Beads were washed with 400 μl of TAP-B4 (50 mM Tris-Cl [pH 7.8], 200 mM NaCl, 1.5 mM MgAc, 1 mM DTT, 2 mM CaCl_2_, 0.15% NP-40) followed by 200 μl of TAP-B5 (50 mM Tris-Cl [pH 7.5], 200 mM NaCl, 1.5 mM MgAc, 0.5mM CaCl_2_). Finally, the proteins were eluted by adding 600 μl of TAP-EB (20 mM Tris-Cl [pH 7.9], 5 mM EGTA) to the beads and incubating for 30 min at room temperature, and were then precipitated with trichloroacetic acid.

A similar, but more stringent, procedure was used to purify the complexes shown in Figures 2A and 4B. The main differences were an increase in salt concentration to 350 mM NaCl during extraction, column binding, and washing and the amount of washes applied to the columns, which were increased to 40 column volumes at each step. In addition, 10% glycerol was present in all buffers.

#### Protein identification

The protein composition of the final fraction resulting from the TAP procedure was determined using Direct Analysis of Large Protein Complexes technology as described previously (Sanders et al. 2002). Briefly, proteins were precipitated and proteolyzed by trypsin. The peptides resulting from the digestion were separated by multidimensional capillary chromatography and subjected to mass spectrometry.

#### Analytical-scale affinity purifications

For coprecipitation assays, we prepared extracts from 150 ml yeast cultures harvested at an OD_600_ of 1.0. Cells were pelleted, washed with PBS, and resuspended in 0.6 ml of TAP-IPB (50 mM Tris [pH 7.8], 150 mM NaCl, 1.5 mM MgAc, 0.15% NP-40, 1 mM DTT, 10 mM NaPPi, 5 mM EGTA, 5 mM EDTA, 0.1 mM Na_3_VO_4_, 5 mM NaF, Complete^TM^ Protease inhibitor cocktail). Acid-washed glass beads were added, and the cells were disrupted mechanically using a bead beater (BioSpec Products, Bartlesville, Oklahoma, United States) for 5 min. Insoluble material after cell disruption was removed by centrifugation in a microfuge at 14,000 rpm for 20 min. The supernatant was incubated with 25 μl of IgG sepharose beads (Amersham Biosciences) for 90 min at 4 °C. Beads were then pelleted and washed three times with 0.6 ml of TAP-IPB. After washing, the beads were resuspended in SDS sample buffer and subjected to SDS PAGE and immunoblotting with anti-HA-Peroxidase antibody (#2 013 819; Roche) and antibodies against Tra1p (a generous gift from J. Workman), Bdf1p (a generous gift from A. Ladurner), and Act1p (a generous gift from D. Drubin).

#### Microarray expression analysis

The strains used for expression analysis were derived from S288c: YM1823 *MATα swr1*Δ*::kanMX4 his3*Δ*1 leu2*Δ*0 ura3*Δ*0 lys2*Δ*0* (obtained from the *MATα* yeast deletion collection; Research Genetics, Huntsville, Alabama, United States) and YM1769 *MATα his3*Δ*1 leu2*Δ*0 ura3*Δ*0 lys2*Δ*0.* Exponentially growing cultures were diluted to OD_600_ 0.1 in yeast extract-peptone-dextrose medium (YPD) (Qbiogene, Carlsbad, California, United States) supplemented with tryptophan and adenine. Each mutant culture was paired with a wild-type (wt) culture placed in an adjacent slot in a shaker. Four such pairs of cultures were grown at 30 °C to OD_600_ 0.8. Cultures were harvested at identical optical densities by vacuum filtration onto nitrocellulose filters (0.45 μm; Millipore, Billerica, Massachuesetts, United States), and snap-frozen in 15 ml conical tubes in liquid nitrogen. Total RNA was extracted as described (http://www.microarrays.org), and mRNA was prepared using oligo-dT coupled to latex beads, using the manufacturer's protocol (Oligotex mRNA Mini Kit; Qiagen, Valencia, California, United States). mRNA was then reverse-transcribed into cDNA.

Microarrays were fabricated as described by DeRisi et al. (1997). Yeast ORFs were amplified using a commercially available primer set (Research Genetics), with yeast genomic DNA as a template. PCR products were verified by gel electrophoresis, precipitated and resuspended in 3X SSC and robotically spotted onto poly-L-lysine-coated glass slides. The exposed poly-L-lysine was then blocked using the succinic anhydride method. Detailed protocols are available at http://www.microarrays.org.

After chemical coupling to Cy5 and Cy3 fluorescent dyes, mutant and wt cDNA samples were mixed and hybridized to microarrays at 63 °C for 12–16 h. Two of the four hybridizations were performed with fluor-reversed samples to avoid artifacts arising from differences in coupling efficiency of the two dyes. After washing and drying, the arrays were scanned on a Genepix 4000B scanner (Axon Instruments, Union City, California, United States) and the images analyzed using Genepix 3.0 software to determine the ratio of median fluorescence intensity (above background) for each spot. After flagging poor quality spots, the ratios were normalized for total signal in the two samples. After filtering the data for dim and uneven spots, genes with at least three good measurements were retained for statistical analysis.

The *swr1*Δ/wt mRNA ratios were analyzed using the SAM (Significance Analysis of Microarrays) statistical package (Tusher et al. 2001) to determine significantly induced or repressed genes. Missing values were estimated using the KNN algorithm with ten nearest neighbors. The analysis was performed with a delta value corresponding to a median false-positive rate less than 1% (Tibshirani et al. 2002). The full dataset is available at http://madhanilab.ucsf.edu/public/swr1.

#### Chromatin immunoprecipitation

ChIP assays were performed and analyzed exactly as described by Meneghini et al. (2003) with the following modifications. DNA derived from the whole cell and pellet fractions was analyzed by real-time PCR and Syber Green fluorescence on an MJ Research (Waltham, Massachusetts, United States) Opticon instrument using DNA derived from whole cell extracts as a standard. Oligonucleotides used correspond to those described by Meneghini et al. (2003) and those in Table S3.

#### Histone acetylation assays

Yeast whole cell extracts were prepared from cells growing in logarithmic phase by glass bead lysis in the presence of trichloroacetic acid. Equal amounts of whole cell extract were subjected to SDS-PAGE and immunoblotting. The antibodies used were directed against tetraacetylated H4 (#05-698; Upstate Biotechnology, Lake Placid, New York, United States), acetylated K5 of H4 (#AHP414; Serotec, Raleigh, North Carolina, United States), acetylated K8 of H4 (Serotec # AHP415), acetylated K12 of H4 (Serotec #AHP416), acetylated K16 of H4 (Serotec #AHP417), and acetylated K9 of H2A (Upstate Biotechnology #07-289).

## Supporting Information

Figure S1 A Fraction of SWR1-Com Subunits Cosedimented Fractions collected from glycerol gradient centrifugations of whole cell extracts containing HA-tagged SWR1-Com subunits (shown on the right) were analyzed by immunoblot with an anti-HA antibody. The gradients were from 10% to 40 % glycerol and 22 0.1-ml fractions were collected in each case, starting at the top (Fraction 1). A percentage of the total cellular pool of all six SWR1-Com subunits that were tested was present in the same fractions, consistent with their association in one complex.
(263 KB PDF).

Figure S2 H2A.Z Was Protected from Degradation by SWR1-Com Three different dilutions of whole cell extracts from wt or *swr1*Δ strains were tested for levels of 3HA-H2A.Z using an anti-HA antibody. Equal amounts of total protein extract were present at each dilution, as seen by the immunoblot with the antibody against Vma1p. The level of H2A.Z in the *swr1*Δ mutant was reduced approximately 2- to 3-fold. This suggested that the SWR1-Com contributed to the stability of H2A.Z, likely by protecting it from protein degradation.
(54 KB PDF).

Table S1 Peptides in the Rvb2-TAP Purification (54 KB PDF).

Table S2 Yeast Strains Used in This Study (95 KB PDF).

Table S3 ChIP Oligo Sequences (60 KB PDF).

### Accession Numbers

The *Saccharomyces* genome database (http://www.yeastgenome.org) accession numbers of the proteins discussed in this paper are actin (SGDID S0001855), Arp4 (SGDID S0003617), Asf1p (SGDID S0003651), Bdf1p (SGDID S0004391), CAF-I (SGDID S0006222), Cse4p (SGDID S0001532), Eaf3p (SGDID S0006227), Epl1p (SGDID S0001870), Esa1p (SGDID S0005770), H2A (SGDID S0002633), H2B (SGDID S0002632), Kap114p (SGDID S0003210), Nap1p (SGDID S0001756), Rvb1p (SGDID S0002598), Rvb2p (SGDID S0003118), Rvb2p (SGDID S0006156), SAS-I HAT (SGDID S0005739), Sir2p (SGDID S0002200), Sir3p (SGDID S0004434), Sir4p (SGDID S0002635), Swc2p (SGDID S0002893), Swc3p (SGDID S0000009), Swc4p (SGDID S0003234), Swc6p (SGDID S0004505), Swc7p (SGDID S0004377), Swi2/Snf2 (SGDID S0005816), Swr1p (SGDID S0002742), Tra1p (SGDID S0001141), Yaf9p (SGDID S0005051), and Yng2p (SGDID S0001132). 

The *Saccharomyces* genome database accession numbers of the genes discussed in this paper are *AAD3* (SGDID S0000704), *HML* (SGDID S0029214), *HMR* (SGDID S0029655), *HTZ1* (SGDID S0005372)*, PRP8* ORF (SGDID S0001208), *YDR359C* (SGDID S0002767), and *YNR074C* (SGDID S0005357). 

The GenBank (http://www.ncbi.nih.gov/Genbank/index.html) accession number of *EAF1* is AY464183.

## Abbreviations

ChIP: chromatin immunoprecipitation
HA: hemagglutinin
HAT: histone acetyltransferase
HU: hydroxyurea
ORF: open reading frame
SWR1-Com: SWR1-Complex
TAP: tandem affinity purification
wt: wild-type
YPD: yeast extract-peptone-dextrose medium
