# Supplementary figures and images for: A Protein Complex Containing the Conserved Swi2/Snf2-Related ATPase Swr1p Deposits Histone Variant H2A.Z into Euchromatin
Source: PLoS Biol. 2004 Mar 23;2(5):e131. doi: 10.1371/journal.pbio.0020131 (PMC374244; doi:10.1371/journal.pbio.0020131)

## Supplementary Fig. 1

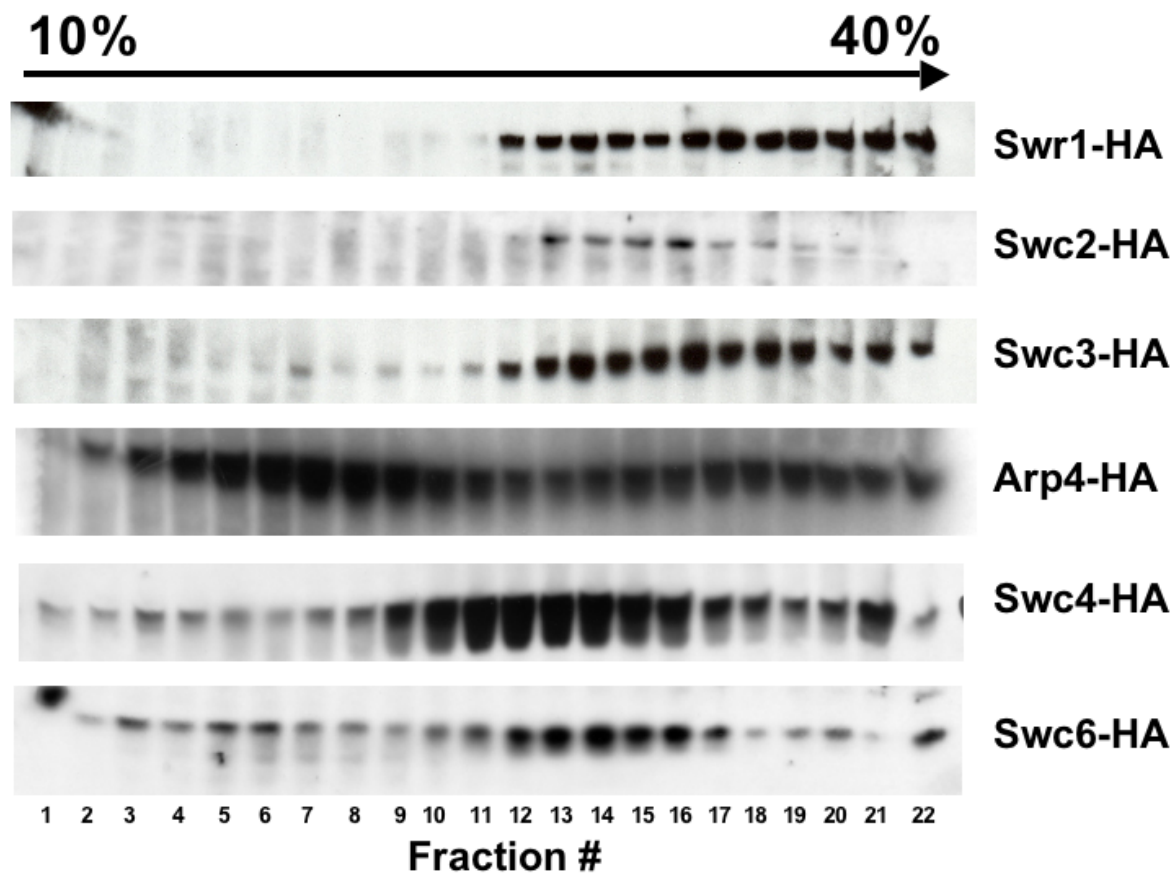

Supplement: Figure S1 — Fractions collected from glycerol gradient centrifugations of whole cell extracts containing HA-tagged SWR1-Com subunits (shown on the right) were analyzed by immunoblot with an anti-HA antibody. The gradients were from 10% to 40 % glycerol and 22 0.1-ml fractions were collected in each case, starting at the top (Fraction 1). A percentage of the total cellular pool of all six SWR1-Com subunits that were tested was present in the same fractions, consistent with their association in one complex. (263 KB PDF). [file pbio.0020131.sg001.pdf]

## Supplementary Fig. 2

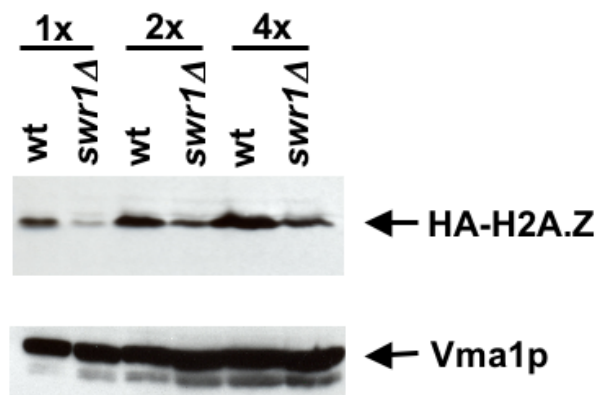

Supplement: Figure S2 — Three different dilutions of whole cell extracts from wt or swr1Δ strains were tested for levels of 3HA-H2A.Z using an anti-HA antibody. Equal amounts of total protein extract were present at each dilution, as seen by the immunoblot with the antibody against Vma1p. The level of H2A.Z in the swr1Δ mutant was reduced approximately 2- to 3-fold. This suggested that the SWR1-Com contributed to the stability of H2A.Z, likely by protecting it from protein degradation. (54 KB PDF). [file pbio.0020131.sg002.pdf]
