# Supplementary material for: A Protein Complex Containing the Conserved Swi2/Snf2-Related ATPase Swr1p Deposits Histone Variant H2A.Z into Euchromatin
Source: PLoS Biol. 2004 Mar 23;2(5):e131. doi: 10.1371/journal.pbio.0020131 (PMC374244; doi:10.1371/journal.pbio.0020131)
Supplement: Table S1 — (54 KB PDF). [file pbio.0020131.st001.pdf]

Supplementary Table 1: Peptides in the Rvb2-TAP purification (SWR1-Com is listed first)

Systematic Name	Name	Mass	Rvb2-TAP
YDR334W	Swr1	174	4
YDR485C	Swc2	91	-
YLR399C	Bdf1	77	-
YAL011W	Swc3	74	-
YJL081C	Arp4	55	2
YGR002C	Swc4	55	2
YPL235W	Rvb2	52	8
YDR190C	Rvb1	50	13
YFL039C	Act1	42	2
YBR231C	Swc5	34	-
YML041C	Swc6	32	1
YNL107W	Yaf9	26	-
YLR385C	Swc7	15	1
YGL150C	Ino80	171	9
YFL013C	Ies1	79	1
YNL215W	Ies2	36	2
YLR052W	Ies3	28	3
