# Supplementary material for: A Protein Complex Containing the Conserved Swi2/Snf2-Related ATPase Swr1p Deposits Histone Variant H2A.Z into Euchromatin
Source: PLoS Biol. 2004 Mar 23;2(5):e131. doi: 10.1371/journal.pbio.0020131 (PMC374244; doi:10.1371/journal.pbio.0020131)
Supplement: Table S2 — (95 KB PDF). [file pbio.0020131.st002.pdf]

Supplementary Table 2
Yeast strains used in this study

Strain	Relevant Genotype	Source*
JRY3009	*MATA ade2-1 can1-100 his3-11 leu2-3,112 trp1-1 ura3-1*	
JRY7720	*MATA ade2-1 can1-100 his3-11 leu2-3,112 trp1-1 ura3-1 bar1*	A. Kirchmaier
JRY7721	JRY7520, *TRP1::ESA1-TAP*++	
JRY7722	JRY7520, *TRP1::YAF9-TAP*	
JRY7723	JRY7520, *TRP1::HTZ1-TAP*	
JRY7724	JRY7520, *kanMX6::SWR1-HA3*	
JRY7725	JRY7520, *kanMX6::SWR1-HA3*, *TRP1::HTZ1-TAP*	
JRY7726	JRY7520, *kanMX6::SWR1-HA3*, *TRP1::HTA1-TAP*	
JRY7727	JRY7520, *kanMX6::SWR1-HA3*, *TRP1::YAF9-TAP*	
JRY7728	JRY7520, *kanMX6::SWC2-HA3*	
JRY7729	JRY7520, *kanMX6::SWC2-HA3*, *TRP1::HTZ1-TAP*	
JRY7730	JRY7520, *kanMX6::SWC2-HA3*, *TRP1::HTA1-TAP*	
JRY7731	JRY7520, *kanMX6::SWC2-HA3*, *TRP1::YAF9-TAP*	
JRY7732	JRY7520, *kanMX6::SWC3-HA3*	
JRY7733	JRY7520, *kanMX6::SWC3-HA3*, *TRP1::HTZ1-TAP*	
JRY7734	JRY7520, *kanMX6::SWC3-HA3*, *TRP1::HTA1-TAP*	
JRY7735	JRY7520, *kanMX6::SWC3-HA3*, *TRP1::YAF9-TAP*	
JRY7736	JRY7520, *kanMX6::SWC4-HA3*	
JRY7737	JRY7520, *kanMX6::SWC4-HA3*, *TRP1::HTZ1-TAP*	
JRY7738	JRY7520, *kanMX6::SWC4-HA3*, *TRP1::HTA1-TAP*	
JRY7739	JRY7520, *kanMX6::SWC4-HA3*, *TRP1::YAF9-TAP*	
JRY7740	JRY7520, *kanMX6::SWC7-HA3*	
JRY7741	JRY7520, *kanMX6::SWC7-HA3*, *TRP1::HTZ1-TAP*	
JRY7742	JRY7520, *kanMX6::SWC7-HA3*, *TRP1::HTA1-TAP*	
JRY7743	JRY7520, *kanMX6::SWC7-HA3*, *TRP1::YAF9-TAP*	
JRY7744	JRY7520, *kanMX6::KAP114-HA3*	
JRY7745	JRY7520, *kanMX6::KAP114-HA3*, *TRP1::HTZ1-TAP*	
JRY7746	JRY7520, *kanMX6::KAP114-HA3*, *TRP1::HTA1-TAP*	
JRY7747	JRY7520, *kanMX6::KAP114-HA3*, *TRP1::YAF9-TAP*	
JRY7748	JRY7520, *TRP1::SWC4-TAP*	
JRY7749	JRY7520, *TRP1::SWR1-TAP*	
JRY7750	JRY7520, *TRP1::RVB2-TAP*	
JRY7751	JRY3009, *yaf9::his5MX6*	
JRY7752	JRY3009, *swr1::his5MX6*	
JRY7753	JRY3009, *swc2::his5MX6*	
JRY7754	JRY3009, *htz1::his5MX6*	
JRY7755	JRY7520, *kanMX6::SWC4-HA3*, *TRP1::SWR1-TAP*	
JRY7756	JRY7520, *kanMX6::SWC4-HA3*, *TRP1::ESA1-TAP*	
JRY7757	JRY7520, *kanMX6::SWC4-HA3*, *TRP1::RVB2-TAP*	
JRY7758	JRY7520, *kanMX6::SWC4-HA3*, *TRP1::INO80-TAP*	
JRY7759	JRY7520, *his5MX6::HTZ1-HA3*	
JRY7760	JRY7520, *his5MX6::HTZ1-HA3, TRP1::SWR1-TAP*	
JRY7761	JRY7520, *his5MX6::HTB1-HA3*	
JRY7762	JRY7520, *his5MX6::HTB1-HA3, TRP1::SWR1-TAP*	
JRY7763	JRY3009, *eaf1::his5MX6*	
JRY7764	JRY7520, *kanMX6::EAF1-HA3*	
JRY7765	JRY7520, *kanMX6::EAF1-HA3, TRP1::HTZ1-TAP*	
JRY7766	JRY7520, *kanMX6::EAF1-HA3, TRP1::YAF9-TAP*	
JRY7767	JRY7520, *kanMX6::EAF1-HA3, TRP1::SWC4-TAP*	
JRY7768	JRY7520, *kanMX6::EAF1-HA3, TRP1::SWR1-TAP*	
JRY7769	JRY7520, *kanMX6::EAF1-HA3, TRP1::ESA1-TAP*	

JRY7770	JRY7520, *his5MX6::SWC3-HA3, kanMX6::SWR1-3HA, TRP1::YAF9-TAP*	
JRY7771	JRY7520, *his5MX6::HTZ1-HA3, kanMX6::SWC4-3HA, TRP1::YAF9-TAP*	
JRY7772	JRY7520, *his5MX6::SWC6-HA3, kanMX6::SWC2-3HA, TRP1::YAF9-TAP*	
YM1769	*MATα his3Δ1 leu2Δ0 ura3Δ0 lys2Δ0*	Research Genetics
YM1823	*MATα swr1Δ::kanMX6 his3Δ1 leu2Δ0 ura3Δ0 lys2Δ0*	Research Genetics
YM1844	*MATalpha his3 leu2 ura3 lys2 HA3-HTZ1::URA3::htz1::kanMX3*	
YM1845	*MATalpha his3 leu2 ura3 lys2 HA3-HTZ1::URA3::htz1::kanMX3 swr1::his5MX6*	

* unless otherwise indicated, all strains were constructed for this work (details available upon request) or were from the laboratory collections. Strains JRY3009-JRY7550 are in the W303 background, strains YM1769-YM1845 are in the S288C background.

[++] this nomenclature indicates the selectable marker, in this case *TRP1*, used to insert the tagged gene, in this case *ESA1-TAP*, at that locus, in this case *ESA1*
