# Supplementary material for: A Protein Complex Containing the Conserved Swi2/Snf2-Related ATPase Swr1p Deposits Histone Variant H2A.Z into Euchromatin
Source: PLoS Biol. 2004 Mar 23;2(5):e131. doi: 10.1371/journal.pbio.0020131 (PMC374244; doi:10.1371/journal.pbio.0020131)
Supplement: Table S3 — (60 KB PDF). [file pbio.0020131.st003.pdf]

Supplementary Table 3

ChIP oligos

Primer set	Oligo sequences
KCC4	AACGCAGTCTTAGTAAAAGG / GTTCTATGGCCCGTGCGCTC
NFS1	TGATAAGCATGGCTCATTGC / GCATACACGAAGGAACTGCT
SGF29	CCAGTACGATCTGCCAACTT / TACAATGCCAACGAGGTCAT
CDC10	AATAGGCTCCCAAGCTTTAG / TTTGATACCATCACGAATCA
SRD1	ACAAAACTAACTGCAAAGTT / AGACTGAAGAAGGAGAATGA
YCR024C	TATTTGTGAGTGGAATAACT / AAAGAAAACGTACCACAATT
SNT1	GCTATTTGGTACTAGGCATC / AAGTCAATAGTAATCCGAAT
BUD31	TCTGCCCGCGCTGGTTCAAA / TGAGGTGAGCTTTGCTAGTC
YCR072C	TCTTCTGTACTGTTATAGCC / GGCTCAACTTCCCAGAGAAG
YCR079W	CTAATGCGTATGGAAGTGCC / GTCCAAGGATCGCACTATCG
ABP1	ATCTATAAACAAACAATCGC / CTTCTCAACGTCTGACCCTG
YCR090C	AACGAAGAACTAAATTTGAA / TATTTCCATGATTCACTGCG
